# Optimizing *in vitro* maturation of oocytes with nitric oxide-loaded microbubbles

**DOI:** 10.3389/fcell.2025.1518226

**Published:** 2025-05-13

**Authors:** Lin Qiu, Qi Xu, Yi Lin, Songxue Wang, Huina Zhang, Yi Liu, Junzhao Zhao, Yingzheng Zhao, Haitao Xi

**Affiliations:** ^1^ Department of Obstetrics and Gynecology, The Second Affiliated Hospital of Wenzhou Medical University, Wenzhou, Zhejiang, China; ^2^ School of Pharmaceutical Sciences, Wenzhou Medical University, Wenzhou, Zhejiang, China; ^3^ Department of Pharmacy, Zhuji People’s Hospital, Zhuji, Zhejiang, China

**Keywords:** nitric oxide, lipid microbubbles, gas-based drug delivery, oocyte, *in vitro* maturation, assisted reproductive technology

## Abstract

**Introduction:**

Nitric oxide (NO) plays a pivotal role in female reproductive processes, yet its clinical translation is limited by its short half-life and rapid systemic clearance. This study aimed to develop a stabilized NO delivery system to enhance oocyte maturation and quality for potential applications in assisted reproductive technologies (ART).

**Methods:**

Lipid-based microbubbles (NO-MBs) were constructed via lyophilization to encapsulate and stabilize therapeutic NO gas. The effects of NO-MBs on in vitro oocyte maturation were evaluated using immature mouse oocytes, with conventional NO donors (e.g., SNP) as controls. Oocyte quality was assessed through Ca2+ levels, mitochondrial membrane potential (MMP), and intracellular reactive oxygen species (ROS). *In vivo* studies further examined oocyte retrieval, fertilization rates, and blastocyst cell apoptosis. Mechanistic investigations focused on the ERK signaling pathway.

**Results:**

The key parameters of the optimized lipid-based microbubbles for NO gas delivery were as follows: particle concentration (2.23 × 10^⁷^ MBs/mL) and diameter (0.919 ± 0.807 μm). NO-MBs demonstrated superior biosafety and efficacy compared to conventional NO donors. *In vitro*, NO-MBs significantly increased the maturation rate of immature mouse oocytes (72.00% vs. 57.77% in controls) while improving oocyte quality, as evidenced by elevated Ca2+ levels, enhanced MMP, and reduced ROS. *In vivo*, NO-MBs enhanced oocyte retrieval and fertilization rates and reduced blastocyst cell apoptosis. Mechanistically, NO-MBs uniquely activated phosphorylated ERK, suggesting ERK pathway involvement in oocyte maturation.

**Discussion:**

These findings highlight NO-MBs as a novel, clinically relevant strategy for targeted NO delivery in reproductive medicine. By optimizing NO release and bioavailability, NO-MBs offer a promising approach to improve oocyte quality and developmental outcomes in ART. Further studies are warranted to explore their broader applications in reproductive health.

## 1 Introduction

Nitric oxide (NO), a gaseous inorganic free radical characterized by a simple structure and high diffusibility, serves as a versatile signaling molecule that regulates a wide range of physiological and pathological processes in mammals (Abu-Soud et al., 2025). Despite its biological half-life of merely ∼2 s, NO rapidly mediates biological signals through interactions with transition metals or thiol groups, while its reactivity also contributes to cytotoxic peroxynitrite formation under oxidative stress (Dao et al., 2020; Lundberg and Weitzberg, 2022). Within the female reproductive system, NO plays pivotal roles in multiple stages of reproduction. It modulates follicular development by regulating granulosa cell apoptosis and steroidogenesis (Masuda et al., 2001), promotes oocyte maturation through the activation of the cGMP-PKG pathway (Bilodeau-Goeseels, 2007), and inhibition of iNOS in rats results in a reduction of ovulation rates by 50% (Awonuga et al., 2023). Additionally, NO is involved in corpus luteum regression, fertilization, embryo implantation, pregnancy maintenance, parturition, and the regulation of menstrual/estrous cycles (Matta et al., 2009; Luo et al., 2021).

*In vitro* maturation (IVM) of oocytes has emerged as a pivotal assisted reproductive technology to rescue immature oocytes—primarily at the germinal vesicle (GV) or metaphase I (MI) stages—by driving their progression to metaphase II (MII) competence under optimized culture conditions (Telfer and Andersen, 2021; Gilchrist and Smitz, 2023). Compared to conventional IVF, IVM significantly reduces the risk of ovarian hyperstimulation syndrome (OHSS), positioning it as a safer alternative for patients with polycystic ovary syndrome (PCOS) or high ovarian response (Siristatidis et al., 2025). However, clinical adoption remains limited due to suboptimal maturation rate (about 60%–70% for MII attainment) and compromised developmental potential of IVM-derived oocytes, attributed to incomplete recapitulation of follicular signaling (Fesahat et al., 2017; Torkashvand et al., 2024). Current strategies to enhance IVM outcomes focus on refining culture systems through supplementation with growth factors [e.g., epidermal growth factor (EGF) and insulin-like growth factor 1 (IGF-1)], antioxidants (e.g., melatonin, resveratrol), and metabolic modulators (Doroudi et al., 2021; Fathi et al., 2021; Hao et al., 2022). NO has emerged as a potential modulator of oocyte maturation. Studies have demonstrated that NO can promote nuclear maturation via cGMP-dependent pathways to accelerate meiotic resumption (Dubeibe et al., 2017), and mitigate oxidative stress by scavenging reactive oxygen species (ROS), thereby preserving cytoplasmic competence in bovine and porcine models (Yang et al., 2024). Additionally, NO has been shown to modulate the expression of key genes involved in oocyte maturation (Cheuquemán et al., 2015), further supporting its role in optimizing IVM. Despite these benefits, NO’s clinical translation faces challenges: its concentration-dependent biphasic effects (pro-maturation at low doses vs. cytotoxic at high levels) (Thaler and Epel, 2003). Concentration-dependent biphasic effects underscore the challenge in utilizing NO for assisted reproductive technologies (ART), particularly in optimizing *in vitro* oocyte maturation (IVM) protocols (Gregg, 2003; Aquila et al., 2011; Rochon and Corti, 2020).

The therapeutic effectiveness of NO is largely dependent on factors such as dosage, duration of exposure, and site of release. To address these challenges, exogenous NO donors—such as organic nitrates (e.g., nitroglycerin), S-nitrosothiols [e.g., sodium nitroprusside (SNP)] and metal-nitrosyl complexes—have been extensively explored for controlled NO delivery (Wang et al., 2002). These donor molecules can be activated to release NO through stimuli such as light, heat, pH, or enzymatic activity, making them the most commonly utilized method for delivering NO in biomedical applications (Wu et al., 2021). Nevertheless, conventional NO donors frequently do not directly supply NO and lack the capacity to target and regulate its release, leading to restricted therapeutic efficacy and possible adverse effects (Yang et al., 2018). Although NO-releasing platform, including scaffolds, particles, and films, have also been reported, various challenges still remain, including limited loading capacity, premature NO leakage, and complex fabrication processes (Alimoradi et al., 2019). Consequently, a delivery system capable of extending the half-life of NO gas, facilitating controlled release of NO, and mitigating the toxic effects of NO medications on healthy tissues holds significant promise for biomedical applications (Paul et al., 2021).

**FIGURE 1:**
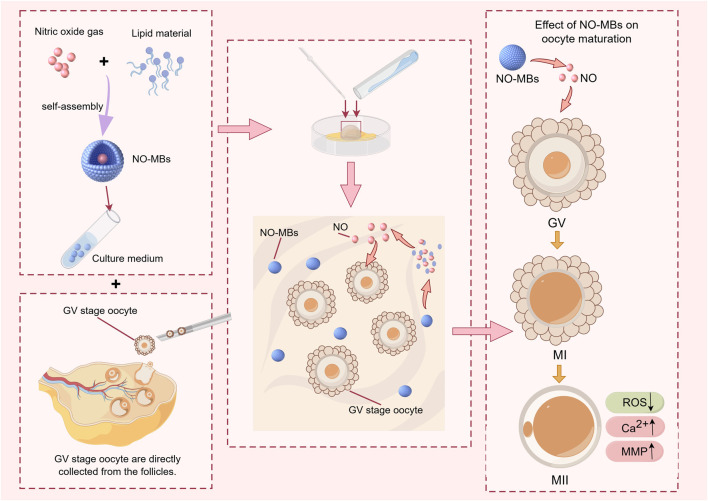
Schematic illustration of Nitric Oxide-loaded microbubbles (NO-MBs) preparation via lyophilization and their role in optimizing oocyte IVM outcomes.

The unique spatiotemporal control offered by NO-loaded microbubbles (NO-MBs) (Fix et al., 2015) positions them as a versatile tool for enhancing ART protocols. As a culture medium additive, NO-MBs could sustain physiological NO levels during IVM of oocytes, circumventing the need for repeated donor supplementation and minimizing oxidative stress. Furthermore, ultrasound-triggered NO release from microbubbles (MBs) could enable localized (Chapla et al., 2022; Kim et al., 2023), synergizing with existing ART workflows. Recent success in microbubble-mediated drug delivery for endometrial receptivity (He et al., 2022) enhancement supports the feasibility of this approach. We hypothesized that NO-MBs could overcome the limitations of traditional NO delivery methods by stabilizing NO in a gas phase to prolong its half-life, achieving controlled release kinetics, and enhancing the developmental competence of immature oocytes by optimizing the microenvironment (Figure 1). To test this hypothesis, we evaluated the feasibility and efficacy of NO-MBs in an oocyte IVM system, focusing on their ability to stabilize NO and promote oocyte maturation. By aligning with clinical infrastructure, NO-MBs offer a scalable strategy to improve oocyte competence and embryo viability in ART.

## 2 Materials and methods

### 2.1 Preparation of MBs and NO-MBs

MBs were fabricated using a lyophilization-based protocol. Hydrogenated soybean phospholipids (HSPC), hydrogenated egg yolk phospholipids (HEPC) and 1,2-distearoyl-sn-glycero-3-phosphocholine (DSPC) were procured from Avite Pharmaceutical Technology Co., Ltd. (Shanghai, China). The lipid components were systematically formulated into six distinct groups. Lipid material (HSPC/HSPC/DSPC) (2.0 mg), Tween-80 (20.0 mg) (Solarbio, Beijing, China), and poloxamer 188 (200.0 mg) (Sigma‒Aldrich, MO, United States) were dissolved in 4.0 mL tert-butyl alcohol (XiLong Science Co., Ltd.). The solution was gradually cooled to complete solidification and subsequently stored at 4 °C overnight. Lyophilization was performed under reduced pressure (5 × 10^−4^ mBar) for 24 h using a three-stage protocol: primary drying at −48°C for 20 h, followed by a secondary drying phase with gradual temperature elevation to 5°C over 4 h. Finally, the powder obtained by freeze-drying was collected in a sterile container. For microbubbles preparation, aliquots of the powder were precisely weighed into vials. The gas exchange process was performed using vacuum-purge cycle displacement with either ambient air or NO gas (99.99%) to generate MBs or NO-MBs, respectively.

### 2.2 Characterization of MBs and NO-MBs

Microbubble suspensions were prepared by injecting 1 mL of phosphate-buffered saline (PBS, pH 7.4) into each vial containing 20.0 mg lyophilized powders. The six distinct formulations of MBs and NO-MBs were reconstituted in PBS and subjected to characterization. Morphological evaluation was conducted using an inverted optical microscope (Eclipse Ti2, Nikon), with representative images captured using NIS-Elements imaging software (v5.21). Quantitative analysis of particle size distribution and concentration was performed using a Multisizer 4e Coulter counter (Beckman Coulter Inc., Brea, CA, USA) with a 30 μm aperture tube. Measurements were conducted in triplicate for each formulation group under controlled temperature (25°C ± 0.5°C). Data acquisition and analysis were performed using the accompanying software (Multisizer 4e Software v3.01) with threshold settings optimized for microbubble detection.

### 2.3 *In vitro* NO release profiling

The NO release profiles from MBs and NO-MBs were quantitatively evaluated using a dialysis method under sink conditions. Briefly, 1.0 mL of NO-MBs suspension (20 mg/mL, 2.23 × 10^7^ MBs/mL) was loaded into a dialysis bag (molecular weight cutoff: 3.5 kDa, Solarbio, Beijing, China) and immersed in 20 mL of phosphate-buffered saline (PBS, pH 7.4) as the receiving medium. Aliquots of the receiving buffer were collected at predetermined time intervals (5, 10, 20, 30, 40, 60, 80, 100, and 120 min) for NO quantification. The detection principle was based on the stoichiometric conversion of released NO to nitrite (NO_2_^−^) via aerobic oxidation (4NO + O_2_ + 2H_2_O → 4NO_2_^−^ + 4H^+^). The nitrite concentration in the receiving buffer was determined spectrophotometrically at 550 nm using a commercial NO assay kit (Solarbio, Beijing, China) according to the manufacturer’s protocol. All measurements were performed in triplicate to ensure data reproducibility.

### 2.4 Cell culture and experimental treatment

Umbilical vein endothelial cells (HUVECs, ATCC^®^ CRL-1730™) were maintained in Dulbecco’s Modified Eagle Medium (DMEM, Gibco, USA) supplemented with 10% (v/v) fetal bovine serum (FBS, Gibco, USA) and 100 U/mL penicillin-streptomycin (Gibco, USA) at 37°C in a humidified 5% CO_2_ incubator (Thermo Scientific, USA). Both murine and human cumulus granulosa cells were cultured in DMEM/F-12 medium (Gibco, USA). Test compounds—including MBs, NO-MBs, and sodium nitroprusside (SNP, Sigma-Aldrich, USA) as positive control—were administered to the culture medium at optimized concentrations and incubated with cells for 12 h under standard culture conditions. All cell culture procedures were performed under aseptic conditions in a Class II biological safety cabinet (Thermo Scientific, USA).

### 2.5 Cytotoxicity assessment of NO-MBs

The cytotoxic effects of MBs and NO-MBs were evaluated using the MTT [3-(4,5-dimethylthiazol-2-yl)-2,5-diphenyltetrazolium bromide] assay in both human cumulus granulosa cells and HUVECs (n = 6 replicates per group). Cells were seeded in 96-well culture plates at a density of 2 × 10^4^ cells/well in 200 μL complete growth medium and allowed to adhere for 12 h at 37°C in a 5% CO_2_ atmosphere. Experimental groups were exposed to: MBs at logarithmic concentrations (1 × 10^5^, 1 × 10^6^, 1 × 10^7^ MBs/mL),NO-MBs at equivalent concentrations Serum-free medium as negative control,1% Triton X-100 as positive control. Following 12 h exposure, 20 μL MTT reagent was added per well and incubated for 4 h at 37°C. The formazan crystals were solubilized with 150 μL DMSO containing 0.01 M glycine buffer (pH 10.5). Absorbance was measured at 570 nm. Cell viability calculated as:

$$\mathrm{Viability}\left(\%\right)=\left(\frac{A_{570,\mathrm{treated}}-A_{570,\mathrm{blank}}}{A_{570,\mathrm{control}}-A_{570,\mathrm{blank}}\;}\right)\times 100$$

### 2.6 Oocyte collection and IVM

The 3–4 weeks female mice were treated according to the guidelines (photoperiod of 12 h and free access to food and water). The mice were injected with PMSG (150 IU/kg). Intraperitoneally 48 h prior to dissection. Mice were anesthetized using chloral hydrate (Jurox Inc.) at 10 mg/kg body weight. The surgical site was disinfected using 70% ethanol, an incision was made on the linea alba, and the ovaries were retrieved, dissected and stored in prewarmed PBS solution. Later, the collected ovaries were sliced using a scalpel blade in M2 media. The released oocytes were then collected in 4-well plates containing 500 µL of M2 solution in each well. Only germinal vesicle oocytes that had three layers of unexpanded cumulus layers and balanced cytoplasm were collected. They were washed three times in M2 solution and then incubated in preprepared IVM media (M199 medium (Gibco, USA) supplemented with 0.1 mM sodium pyruvate, 75 mIU/mL FSH, 10% bovine serum albumin (Gibco, USA) and 1% penicillin-streptomycin and incubated) for maturation. Each COC was placed in a 10 µL droplet of IVM media, covered with mineral oil and incubated at 37°C and 5% CO_2_ for predefined time periods; 18 h.

### 2.7 Detection of NO levels in oocytes

The NO levels in the oocytes were measured using the NO-sensitive fluorescent probe DAF-FM DA (Beyotime Biotechnology Inc.). Oocytes of each group were loaded with 5 µM DAF-FM DA at 37 °C for 30 min and then washed with PBS to remove the surface fluorescence. The intensity of the fluorescence in the whole oocyte was measured by laser scanning microscopy (LSM 800, Zeiss, Germany). The parameters used for image acquisition were similar for all examined oocytes. The experiments were independently repeated 3 times.

### 2.8 Detection of ROS levels in oocytes

The ROS levels in IVM MII oocytes were measured using the ROS-sensitive fluorescent probe DCFH-DA (Beyotime Biotechnology Inc.). Oocytes of each group were loaded with 5 µM DCFH-DA at 37°C for 30 min and then washed with PBS to remove the surface fluorescence. The intensity of the fluorescence in the whole oocyte was measured by laser scanning microscopy (LSM 800, Zeiss, Germany). The parameters used for image acquisition were similar for all examined oocytes. The experiments were independently repeated 3 times.

### 2.9 Mitochondrial function assay in oocytes

The mitochondrial membrane potential (MMP) of the IVM MII oocytes was measured using JC-1 staining (Mitochon-Drial membrane potential assay kit, Abbkine Scientific Co., Wu Han, China). Briefly, oocytes from each group were exposed to JC-1 at 37°C for 20 min and then washed with PBS to remove surface fluorescence. The intensity of the fluorescence in the whole oocyte was measured by laser scanning microscopy (LSM 800, Zeiss, Germany). The parameters used for image acquisition were similar for all examined oocytes. The experiments were independently repeated 3 times.

### 2.10 Detection of calcium levels in oocytes

The calcium levels in IVM MII oocytes were measured using the Ca^2+^-sensitive fluorescent probe Fluo-4 AM (Beyotime Biotechnology Inc.). Oocytes from each group were loaded with 5 µM Fluo-4 AM at 37°C for 30 min and then washed with PBS to remove surface fluorescence. The intensity of the fluorescence in the whole oocyte was measured by laser scanning microscopy (LSM 800, Zeiss, Germany). The parameters used for image acquisition were similar for all examined oocytes. The experiments were independently repeated 3 times.

### 2.11 Collection of mouse zygotes and embryo culture

Four-week-old female ICR mice were divided into four groups: MBs, NO-MBs, NO-MBs+L-NAME, and a control group (normal saline). MBs and NO-MBs were dissolved in normal saline to achieve a dose of 60 mg/kg, while the NO-MBs+L-NAME group received NO-MBs (60 mg/kg) combined with 10 mg/kg L-NAME. All groups received daily intraperitoneal injections for 7 days. Ovarian hyperstimulation was induced with PMSG (10 IU/mouse) followed by hCG (10 IU/mouse) 46–48 h later. All groups received hormone injections at synchronized time points. Immediately after hCG administration, females were cohoused with mature male mice. Vaginal plugs were checked 16–18 h post-mating, and plug-positive females were humanely euthanized simultaneously across all groups (n = 3 per group). Zygotes were collected from the ampulla of both fallopian tubes 20 h post-hCG injection (n = 40–60 zygotes per group), with all groups processed in parallel within a 30-min window to eliminate temporal confounding. After hyaluronidase-mediated granulosa cell removal, zygotes were cultured in pre-equilibrated embryo medium under oil at 37°C with 5% CO_2_. Developmental stages (2-cell, 4-cell, morula, blastocyst) were assessed at standardized time points respectively. The whole procedure was repeated three times.

### 2.12 Western blotting

Protein quantification was performed using a bicinchoninic acid (BCA) assay. Equal amounts of protein lysates (20 μg per lane) were separated by 12% sodium dodecyl sulfate–polyacrylamide gel electrophoresis and transferred onto polyvinylidene difluoride membranes for Western blot analysis. After blocking with 5% bovine serum albumin for 2 h, the membranes were then incubated with anti-eNOS (1:500 dilution) (Affinity Biosciences), anti-iNOS (1:200 dilution) (Affinity Biosciences), anti-ERK 1/2 (1:1,000 dilution), and anti-p-ERK1/2 (1:1,000 dilution) antibodies as primary antibodies at 4°C overnight. The membranes were washed before incubation with HRP-conjugated goat anti-IgG (H+L) secondary antibody (1:10,000 dilution) for 1 h at room temperature. The relative band density was calculated using ImageJ software, and an antibody against GAPDH was used as an internal control.

### 2.13 Statistical analysis

The data are presented as the mean ± standard deviation (SD) and were analyzed using GraphPad Prism 8.0 (GraphPad Software, USA). Significant differences between two groups were determined by an unpaired Student’s t-test, while multiple comparisons were assessed by one-way ANOVA followed by Tukey’s *post hoc* test. A *p*-value <0.05 was considered statistically significant.

## 3 Results

### 3.1 Optimization of lipid microbubble formulations for NO gas encapsulation

**FIGURE 2:**
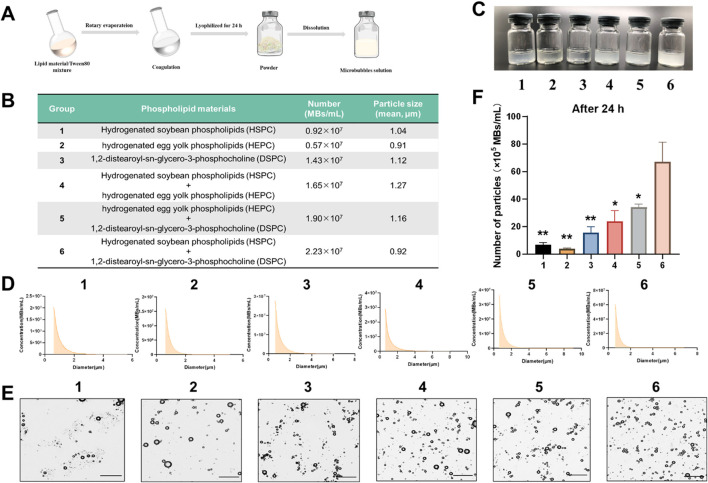
Preparation and characterization of lipid microbubbles. **(A)** Schematic diagram of the microbubble preparation process. **(B)** Composition, particle number and mean particle size of groups 1–6. **(C)** Macroscopic appearance of Groups 1–6 (100 mg MBs in 1 mL PBS). **(D)** Size distribution profiles of Groups 1–6. **(E)** Optical microscopy images of Groups 1–6 (scale bar = 10 μm). **(F)** Particle number retention after 24 h in solution. Data are presented as mean ± SD; *P < 0.05, **P < 0.01vs. Group 6.

Six lipid formulations were systematically evaluated for microbubble stability and gas encapsulation potential. Single-component systems (Group 1: HSPC; Group 2: HEPC; Group 3: DSPC) and combinatorial formulations (Group 4: HSPC+HEPC; Group 5: HEPC+DSPC; Group 6: HSPC+DSPC) were prepared via lyophilization (Figures 2A, B) and characterized.

All formulations exhibited homogeneous curd-like suspensions, with visually distinct turbidity levels observed across the groups (Figure 2C). Particle size distribution and concentration analysis revealed that combination groups exhibited higher particle numbers compared to single-component groups (Figures 2B, D). Among these, Group 6 (HSPC + DSPC) demonstrated the highest particle concentration (2.23 × 10^7^ MBs/mL). Optical microscopy analysis revealed distinct variations in particle density across the groups, with Group 6 demonstrating both higher particle concentration and superior size uniformity compared to other formulations (Figure 2E). To assess stability, the particle numbers of 20 mg/mL microbubble solutions were measured after 24 h at room temperature. Group 6 maintained the highest particle number [(67.00 ± 14.38) ×10^5^ MBs/mL, *p* < 0.05 vs. all groups (Figure 2F)], indicating superior stability. In summary, the combination of HSPC and DSPC was used as the lipid material for microbubbles.

### 3.2 Construction and characterization of NO-loaded microbubbles

**FIGURE 3:**
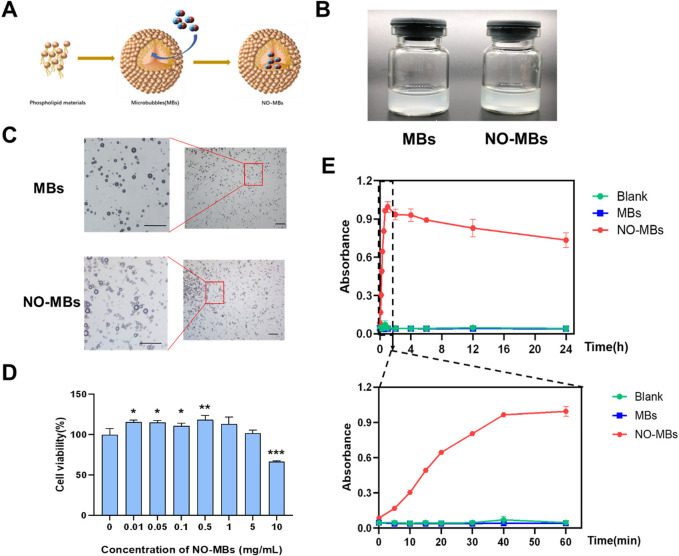
Fabrication and functional evaluation of NO-MBs. **(A)** Schematic illustration of NO-MB construction via lipid self-assembly and gas encapsulation. **(B)** Macroscopic comparison of blank microbubbles (MBs, left) and NO-MBs (right) suspended in PBS (100 mg/mL). **(C)** Optical micrographs of MBs and NO-MBs (scale bar = 10 μm). **(D)** HUVEC viability assay following NO-MB exposure, indicating concentration-dependent cytocompatibility.; *P < 0.05, **P < 0.01, **P < 0.001 vs. 0 mg/mL control. **(E)** NO release kinetics quantified by chemiluminescence: Burst release phase (0–60 min, lower panel) and sustained release profile (0–24 h, upper panel) at 37°C.

Based on preliminary screening of carrier materials for NO gas delivery (Section 3.1), NO-loaded microbubbles (NO-MBs) were constructed and characterized for stability, biological safety, and functional efficacy. The NO loading methodology is illustrated in Figure 3A. Both blank microbubbles (MBs) and NO-MBs exhibited homogeneous colloidal suspensions (100 mg/mL in PBS) with equivalent macroscopic phase behavior (Figure 3B). Microscopic examination revealed polydisperse spherical particles with no evidence of aggregation or structural deformation post-NO loading (Figure 3C).

Cytotoxicity assessment demonstrated excellent biocompatibility of NO-MBs across a concentration range of 0–5 mg/mL (Figure 3D). Notably, low-dose exposure (≤0.5 mg/mL) exhibited a mild proliferative effect on HUVECs (viability = 118.38 ± 55.63% vs. control, p < 0.01), potentially attributable to NO-mediated endothelial cell activation.

*In vitro* release profiles of NO-MBs were quantified using chemiluminescence detection (Figure 3E). Blank MBs showed baseline NO levels, confirming encapsulation specificity. NO-MBs exhibited a biphasic release profile: an initial burst phase (0–60 min) achieving maximal NO concentration (21.70 ± 0.17 μM), followed by sustained release maintaining therapeutic levels (16.6 ± 1.13 μM) over 24 h. These results demonstrated that NO-MBs enable stable, sustained, and biologically safe NO delivery.

### 3.3 NO-MBs supplementation enhances the maturation of immature oocytes

**FIGURE 4:**
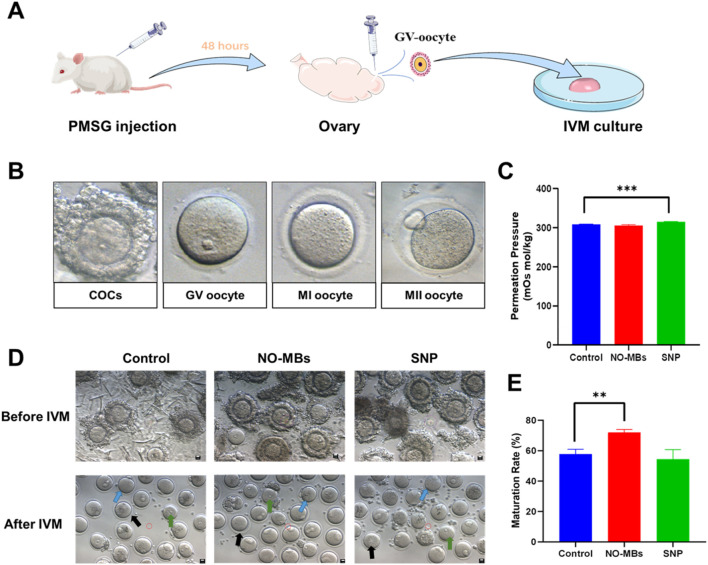
Functional of NO-MB in oocyte maturation assays. **(A)** Experimental workflow for isolating GV stage oocytes from superovulated mice. **(B)** Schematic representation of cumulus-oocyte complex (COC) morphology and developmental stages: GV (germinal vesicle), MI (metaphase I), and MII (metaphase II). **(C)** Comparative analysis of permeation pressure in control, NO-MB-treated, and SNP-treated groups. Data expressed as mean ± SD (n = 3 independent replicates). **(D)** Phase-contrast microscopy images of oocytes cultured under different conditions: MII (blue arrows), MI (black arrows), and GV (green arrows) stages (scale bar = 10 μm) **(E)** Quantitative analysis of oocyte nuclear maturation rates. Control group: oocytes were incubated in IVM culture medium; NO-MBs group: oocytes were incubated in IVM culture medium with NO-MBs; SNP group: oocytes were incubated in IVM culture medium with SNP. **P < 0.01, ***P < 0.001 vs. control. Data represent mean ± SEM from three independent experiments (n = 35–70 oocytes/group).

To evaluate the reproductive potential of NO-MBs, GV-stage oocytes were isolated from 3-4-week-old ICR mice following superovulation (10 IU PMSG, 46–48 h) and cultured in IVM medium supplemented with NO-MBs or SNP. Oocyte retrieval was performed via ovarian dissection post-cervical dislocation (Figure 4A). And as shown in Figure 4B, morphological analysis of oocytes at different developmental stages revealed distinct characteristics.

Osmolarity measurements provided critical insights into culture conditions: While NO-MBs supplementation maintained physiological osmolarity (306.00 ± 2.00 mOsm/kg vs. control 308.67 ± 0.58 mOsm/kg, P = 0.21), SNP treatment significantly elevated medium osmolarity (315.00 ± 1.00 mOsm/kg, P < 0.001 vs. control) (Figure 4C), potentially contributing to its detrimental effects on oocyte maturation. All of the GV oocytes were collected and divided into three groups (Control, NO-MBs and SNP) in the subsequent IVM experiments. As shown in Figure 4D, morphological evaluation of oocytes after IVM culture revealed superior cytoplasmic quality in the NO-MBs-treated group compared to other groups. Quantitative analysis demonstrated that NO-MBs supplementation significantly improved the maturation rate of immature oocytes (72.00%) compared to the control group (57.77%, P = 0.003) (Figure 4E). In contrast, the NO donor SNP treatment resulted in a reduced maturation rate relative to the control group.

### 3.4 NO-MBs supplementation improved the quality of IVM-MII oocytes

Microscopic observation revealed that oocytes in the NO-MBs group exhibited superior morphological characteristics compared to the other groups. To further evaluate the quality and developmental potential of these oocytes, we analyzed several key biomarkers associated with oocyte competence.

**FIGURE 5:**
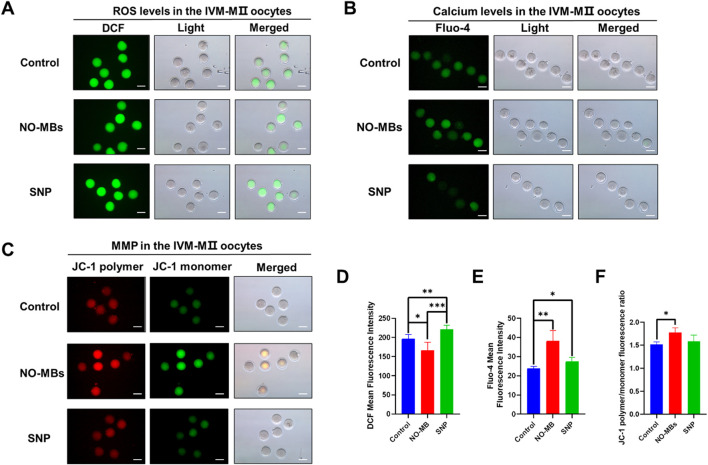
NO-MBs improve the quality of oocytes. **(A)** Representative images of intracellular reactive oxygen species (ROS) levels in IVM-derived MII oocytes. **(B)** Intracellular calcium (Ca^2+^) distribution in IVM-MII oocytes. **(C)** Mitochondrial membrane potential (MMP) assessed by JC-1 staining in IVM-MII oocytes. Quantitative analysis of fluorescence intensity: **(D)** ROS levels: NO-MBs vs. Control *P < 0.05; SNP vs. Control **P < 0.01; SNP vs. NO-MBs ***P < 0.001. **(E)** Ca^2+^ concentrations: NO-MBs vs. Control **P < 0.01; SNP vs. Control *P < 0.05. **(F)** MMP red/green ratio: NO-MBs treatment significantly increased mitochondrial polarization (*P < 0.05) Images acquired using inverted fluorescence microscopy (Scale bar = 100 µm). Data represent mean ± SEM from three independent experiments (n = 5–8 oocytes/group).

Intracellular reactive oxygen species (ROS), critical for spindle formation, chromosome alignment, and oocyte maturation (Jiao et al., 2021), were significantly reduced by NO-MBs treatment compared to controls (n = 7; P < 0.05). In contrast, SNP-treated oocytes (n = 6) exhibited elevated ROS levels versus both control (P < 0.01) and NO-MBs groups (P < 0.001) (Figures 5A, D).

Calcium (Ca^2+^) signaling, essential for meiotic resumption and germinal vesicle breakdown (Deguchi et al., 2015), was enhanced by NO-MBs (n = 8; P < 0.01 vs. control), while SNP treatment (n = 5) suppressed Ca^2+^ levels (P < 0.05 vs. control) (Figures 5B, E). This suggests NO-MBs promote Ca^2+^ oscillations, facilitating meiotic resumption and cortical granule exocytosis—key mechanisms for preventing polyspermy.

Mitochondrial function, assessed via membrane potential (MMP) using JC-1 fluorescence, revealed significantly increased MMP in NO-MBs-treated oocytes (Figures 5C, F). JC-1 aggregates (red fluorescence, ΔΨm >140 mV) increased relative to monomers (green fluorescence, ΔΨm <100 mV), indicating enhanced mitochondrial polarization critical for energy production and developmental competence (Kirillova et al., 2021).

### 3.5 NO-MBs enhance oocyte quality through NO signaling and ERK1/2 pathway activation

**FIGURE 6:**
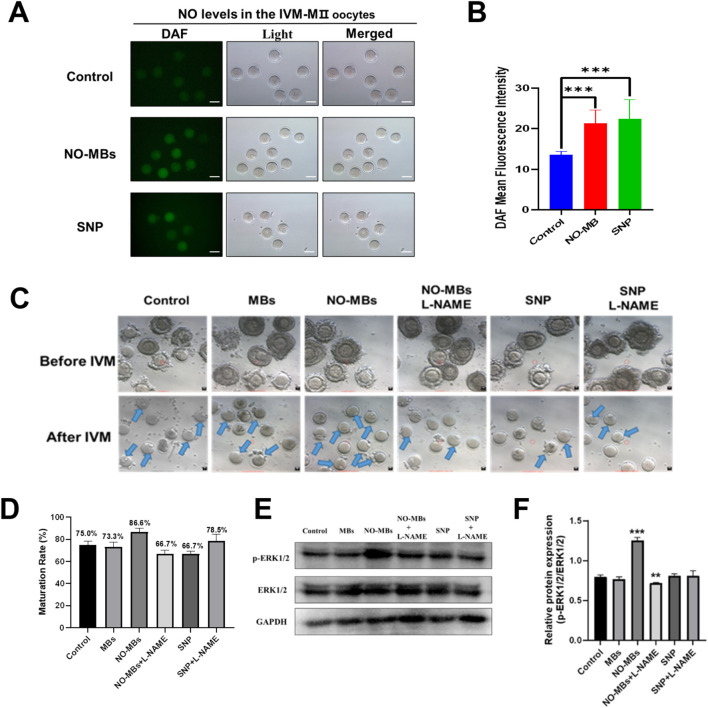
NO-MBs enhance oocyte quality through NO signaling and ERK1/2 pathway activation. **(A)** Representative fluorescence micrographs of IVM-MII oocytes stained with DAF-FM for nitric oxide (NO) detection. Images were acquired using an inverted fluorescence microscope (Scale bar = 100 µm). **(B)** Quantification of NO fluorescence intensity across groups. NO-MBs vs. Control: ***P < 0.001; SNP vs. Control: **P < 0.01. **(C)** Morphological progression of oocytes during IVM: GV-stage (immature, upper) vs. MII-stage (mature, lower) (Scale bar = 10 µm). **(D)** Bar chart of oocyte maturation rates in each group, NO-MBs vs. Control: **P < 0.01. Data represent mean ± SEM from three independent experiments (n = 20–40 oocytes/group). **(E)** Western blot analysis of ERK1/2 and phosphorylated ERK1/2 (p-ERK1/2) protein expression in oocytes from each treatment group. **(F)** Relative expression of p-ERK1/2 normalized to total ERK1/2, indicating pathway activation status. (n = 6–10 oocytes/group for fluorescence analysis).

To confirm the effect of NO-MBs on intracellular NO levels of oocytes, we measured the concentration of NO in the IVM-MII oocytes of each group. Quantitative analysis revealed that both NO-MBs and SNP treatments significantly enhanced NO generation compared to controls (n = 10). Specifically, mean fluorescence intensity increased in the NO-MBs group (n = 7) and SNP group (n = 6) (P < 0.001; Figures 6A, B), confirming NO-MBs as an effective NO donor in oocytes.

To determine whether NO-MBs promote maturation through nitric oxide synthase (NOS)-mediated NO synthesis, we employed L-NAME, a specific NOS inhibitor. Immature oocytes were divided into six experimental groups: Control, MBs, NO-MBs, NO-MBs + L-NAME, SNP, SNP + L-NAME (Figure 6C). Maturation rates demonstrated a NOS-dependent mechanism for NO-MBs: NO-MBs significantly enhanced maturation (86.6% vs. 75% control; P < 0.01); L-NAME abolished this effect (66.7% vs. 86.6%; P < 0.01), even reducing maturation below control levels. In contrast, L-NAME paradoxically improved maturation in SNP-treated oocytes (78.5% vs. 66.7%; P < 0.05; Figure 6D). These findings reveal the dual role of NO in oocyte development, where endogenous (NOS-mediated) and exogenous NO sources exert distinct biological effects.

Western blot analysis of ERK1/2 phosphorylation status provided mechanistic insights: NO-MBs uniquely enhanced p-ERK1/2 expression (vs. control; P < 0.01). Total ERK1/2 levels remained stable across groups (NS). Phosphorylation ratio (p-ERK/ERK) was highest in the NO-MBs group (Figures 6E, F). This selective ERK activation suggests that NO-MBs promote maturation through both NO release and downstream MAPK signaling modulation. In summary, NO-MBs enhance oocyte maturation through: sustained NO release, NOS-dependent endogenous NO synthesis, selective activation of ERK signaling pathway. These findings establish NO-MBs as a multifaceted modulator of oocyte quality, offering potential applications in assisted reproductive technologies.

### 3.6 *In vivo* administration of NO-MBs enhances reproductive outcomes

**FIGURE 7:**
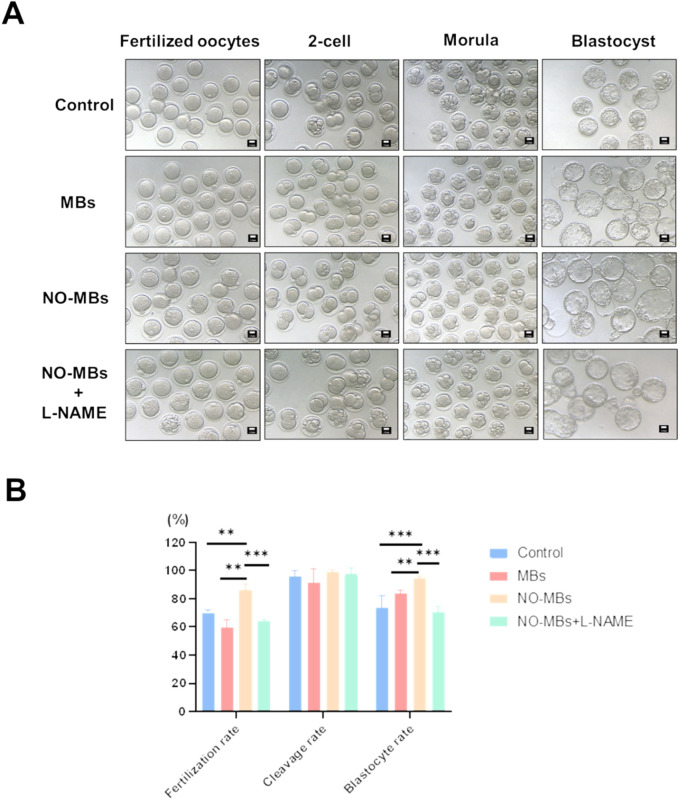
NO-MBs enhance *in vivo* embryonic development. **(A)** Representative light microscopy images documenting embryonic progression at key developmental stages: fertilization: pronuclear formation (18–20 h post-IVF), cleavage: 2-cell stage (24–26 h), blastocyst: expanded cavity formation (96–120 h), Scale bar = 10 μm. **(B)** Comparison of embryo fertilization rate, cleavage rate and blastocyst rate. *P < 0.05, **P < 0.01, ***P < 0.001, vs. control.

To comprehensively evaluate the safety and efficacy of NO-MBs on embryonic development, we conducted a systematic *in vivo* study in female mice. Following 1 week of continuous intraperitoneal administration (5 mg/kg/day), oocytes were retrieved and subjected to *in vitro* fertilization (IVF) and embryo culture. NO-MBs treatment significantly improved developmental parameters compared to controls: fertilization rate: 86.32% (NO-MBs) vs. 70.04% (Control) (**P < 0.01). Blastocyst formation rate: 94.42% vs. 73.48% (***P < 0.001) (Figures 7A, B).

**FIGURE 8:**
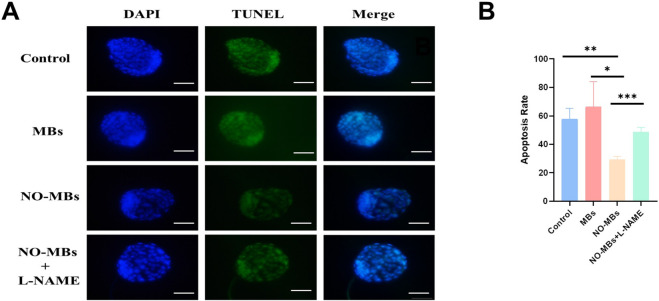
NO-MBs Enhance Blastocyst Viability by Reducing Apoptosis. **(A)** Apoptosis assessment in blastocysts using dual fluorescence staining: DAPI (blue): Nuclear counterstain for total cell count, TUNEL (green): Apoptotic cells with DNA fragmentation. Scale bar = 50 μm. **(B)** Quantitative analysis of apoptosis rates. *P < 0.05, **P < 0.01, ***P < 0.001. n = 15 blastocysts/group from three independent replicates.

TUNEL/DAPI double staining was used to detect the apoptosis rate of blastocyst cells. The results showed that the apoptosis rate of blastocyst cells in the NO-MBs (71.0%) intraperitoneal injection group was significantly lower than that in the control group (86.5%) and MBs injection group (76.2%) (Figures 8A, B).

## 4 Discussion

In this study, we successfully engineered microbubbles (MBs) using an optimized lipid composition comprising a mixture of HSPC and DSPC. The NO-loaded MBs (NO-MBs) were specifically designed to facilitate the controlled delivery of therapeutic nitric oxide (NO) gas into the oocyte *in vitro* maturation (IVM) culture system. Through comprehensive *in vivo* and *in vitro* experiments, we demonstrated that NO-MBs enable stable and sustained release of NO, while maintaining high biosafety. Our findings indicate that supplementation with NO-MBs significantly enhances oocyte maturation and improves the quality of mature oocytes. This beneficial effect appears to be mediated, at least in part, through the activation of ERK signaling pathways, which play a critical role in regulating meiotic progression and cytoplasmic maturation.

Over the past decades, nitric oxide (NO) has emerged as a critical regulator of human reproduction, with studies highlighting its role in folliculogenesis, oocyte maturation, and embryo development (Thaler and Epel, 2003). The clinical application of NO has been limited by its short half-life (<2 s in physiological conditions) and narrow therapeutic window, which can lead to paradoxical effects depending on concentration and timing. To address these challenges, lipid-based delivery systems have been explored for their ability to control NO release kinetics and target specific biological compartments (Hernot and Klibanov, 2008). In this study, we engineered a novel microbubble platform by optimizing the lipid composition (HSPC + DSPC hybrid membrane), which demonstrated superior stability and sustained NO release without requiring ultrasound activation—a significant advancement over conventional microbubble technologies.

The dual nature of NO in female reproduction has been well-documented, with its effects ranging from promoting meiotic resumption at low concentrations (0.1 μM) to inhibiting germinal vesicle breakdown (GVBD) at higher levels (1–500 μM) (Bilodeau-Goeseels, 2007). This concentration-dependent duality underscores the importance of precise NO delivery. Our data reveal that NO-MBs achieve a controlled release profile, reaching a maximum concentration within 6 h and maintaining therapeutic levels for up to 24 h. This sustained release not only stabilizes NO’s half-life but also ensures that its concentration remains within the physiological range, thereby avoiding the detrimental effects associated with supraphysiological NO levels.

The assessment of ROS, Ca^2+^, and MMP dynamics in IVM-MII oocytes revealed fundamental insights into NO-MBs’ protective mechanisms during oocyte maturation. ROS, while essential for nuclear maturation through oxidative phosphorylation (Sahoo et al., 2022), must be tightly regulated to prevent oxidative damage to critical cellular components (Prasad et al., 2016; Wang et al., 2021). Our findings demonstrate that NO-MBs effectively maintained this balance, reducing intracellular ROS levels compared to controls (P < 0.01) and SNP-treated oocytes (P < 0.001). Equilibrium between ROS production and antioxidative capacity is critical to oocyte development and quality. Calcium signaling, a crucial second messenger system in oocyte maturation (Martin et al., 2017). The level of intracellular Ca^2+^ is also a manifestation of mitochondrial function. Mitochondria can store Ca^2+^ and maintain calcium homeostasis in an oocyte (Zhang et al., 2022). MMP is also one of the major indicators reflecting mitochondrial function. A positive relationship between mitochondrial functionality and oocyte development has been established (Tavares et al., 2021). It was found that the Ca^2+^ levels and MMP of the oocytes in the NO-MBs group were higher than those in the control group or SNP group; the intracellular ROS level was lower than that in the other two groups. The above results indicated that the quality of oocytes in the NO-MBs group was well protected during IVM culture.

In addition, we observed that the addition of NO-MBs can increase the fertilization rate and blastocyst formation rate and decrease the blastocyst cell apoptosis rate in mouse oocyte *in vitro* fertilization and embryo culture trials. It has been reported that with the increase in the concentrations of L-NAME, SNP or 8-Br-cGMP (inhibitors of NOS), the blastocyst hatching rate was significantly lowered (Pan et al., 2015). The level of NO metabolism is also related to the successful formation of blastocysts before implantation (Lipari et al., 2009). These publications are consistent with our results, showing that NO may influence the blastocyst formation rate and blastocyst quality.

In our research, we found that NO-MBs can activate the ERK pathway. Previous studies have shown that the ERK pathway is involved in the process of oocyte maturation (Nath et al., 2018). Mechanistically, the sustained release of nitric oxide (NO) from NO-MBs was found to activate the ERK signaling pathway, as evidenced by elevated phosphorylated ERK (p-ERK) levels in cumulus-oocyte complexes. This aligns with previous reports that ERK activation in cumulus cells is indispensable for gonadotropin-induced germinal vesicle breakdown (GVBD) and granulosa cell proliferation (Sha et al., 2017). Some molecules, such as the methionine adenosyltransferase 2β gene (Mat2b), have been reported to participate in oocyte maturation by regulating ERK (An et al., 2020). In this paper, we confirmed that NO-MBs can promote the protein level of p-ERK, however, the absence of p-ERK upregulation in other treatment groups (e.g., free NO donors) suggests that the delivery modality critically determines biological outcomes. While conventional NO donors generate acute concentration spikes that may trigger feedback inhibition or oxidative stress, NO-MBs provide gradual, localized NO release through ultrasound-triggered microbubble collapse. This spatiotemporal control likely preserves ERK activation thresholds necessary for pro-maturation signaling while avoiding cytotoxic nitric oxide synthase suppression or premature pathway desensitization Therefore, we speculate that NO-MBs can effectively and continuously release NO and promote oocyte development through the ERK signaling pathway.

While our findings highlight the therapeutic potential of NO-MBs, several limitations should be acknowledged. First, the current study utilized a murine model, which may not fully recapitulate the physiological complexity of human reproductive systems. For instance, differences in follicular dynamics and hormonal regulation between rodents and humans could influence NO-MBs’ translatability to clinical IVF protocols. Second, the sample size was relatively small, potentially limiting statistical power to detect subtle effects. Third, the mechanism by which NO-MBs activate ERK signaling remains partially elucidated; whether this occurs through direct NO-mediated post-translational modifications or indirect paracrine crosstalk requires further investigation. Fourth, the long-term safety and off-target effects of NO-MBs *in vivo* remain to be systematically evaluated. To address these limitations, future studies will prioritize: (1) validation in non-human primate models to better approximate human reproductive physiology, (2) large-scale, multi-center clinical trials to assess efficacy and safety in human ART protocols, and (3) mechanistic investigations into NO-MB interactions to further optimize therapeutic outcomes. Addressing these limitations will accelerate the translation of NO-MBs into clinical practice.

## 5 Conclusion

In conclusion, our study demonstrates that nitric oxide-loaded microbubbles (NO-MBs) represent as a breakthrough platform for spatiotemporally controlled NO delivery in reproductive medicine. NO-MBs enable precise and sustained NO release under non-ultrasonic conditions, addressing critical limitations of conventional NO donors. Our findings demonstrate that NO-MBs significantly enhance mouse oocyte maturation and embryo development through activation of the ERK signaling pathway. Our research positions NO-MBs as a clinically viable strategy to improve IVF outcomes, potentially elevating fertilization rates and embryo viability in patients with poor ovarian response.

## Data Availability

The raw data supporting the conclusions of this article will be made available by the authors, without undue reservation.
